# Gestation-dependent increase in cervicovaginal pro-inflammatory cytokines and cervical extracellular matrix proteins is associated with spontaneous preterm delivery within 2 weeks of index assessment in South African women

**DOI:** 10.3389/fimmu.2024.1377500

**Published:** 2024-08-06

**Authors:** Emmanuel Amabebe, Nadia Ikumi, Ally Oosthuizen, Priya Soma-Pillay, Mushi Matjila, Dilly O. C. Anumba

**Affiliations:** ^1^ Division of Clinical Medicine, University of Sheffield, Sheffield, United Kingdom; ^2^ Division of Anatomical Pathology, Department of Pathology, University of Cape Town, Cape Town, South Africa; ^3^ Department of Obstetrics and Gynaecology, University of Cape Town, Cape Town, South Africa; ^4^ Department of Obstetrics and Gynaecology, University of Pretoria, Pretoria, South Africa

**Keywords:** preterm birth, cervicovaginal fluid, inflammation, cytokine, extracellular matrix remodelling

## Abstract

**Introduction:**

Inflammation-induced remodelling of gestational tissues that underpins spontaneous preterm birth (sPTB, delivery < 37 weeks’ gestation) may vary by race and context. To explore relationships between markers of these pathological processes, we (a) characterised the cervicovaginal fluid (CVF) cytokine profiles of pregnant South African women at risk of PTB; (b) determined CVF matrix-metalloproteinase-9 (MMP-9) and its regulator tissue inhibitor of metalloproteinase-1 (TIMP-1); and (c) explored the predictive potential of these markers for sPTB.

**Method of study:**

The concentrations of 10 inflammatory cytokines and MMP-9 and TIMP-1 were determined by ELISA in CVF samples from 47 non-labouring women at high risk of PTB. We studied CVF sampled at three gestational time points (GTPs): GTP1 (20–22 weeks, n = 37), GTP2 (26–28 weeks, n = 40), and GTP3 (34–36 weeks, n = 29) and analysed for changes in protein concentrations and predictive capacities (area under the ROC curve (AUC) and 95% confidence interval (CI)) for sPTB.

**Results:**

There were 11 (GTP1), 13 (GTP2), and 6 (GTP3) women who delivered preterm within 85.3 ± 25.9, 51.3 ± 15.3, and 11.8 ± 7.5 (mean ± SD) days after assessment, respectively. At GTP1, IL-8 was higher (4-fold, p = 0.02), whereas GM-CSF was lower (~1.4-fold, p = 0.03) in the preterm compared with term women with an average AUC = 0.73. At GTP2, IL-1β (18-fold, p < 0.0001), IL-8 (4-fold, p = 0.03), MMP-9 (17-fold, p = 0.0007), MMP-9/TIMP-1 ratio (9-fold, p = 0.004), and MMP-9/GM-CSF ratio (87-fold, p = 0.005) were higher in preterm compared with term women with an average AUC = 0.80. By contrast, IL-10 was associated with term delivery with an AUC (95% CI) = 0.75 (0.55–0.90). At GTP3, IL-1β (58-fold, p = 0.0003), IL-8 (12-fold, p = 0.002), MMP-9 (296-fold, p = 0.03), and TIMP-1 (35-fold, p = 0.01) were higher in preterm compared with term women with an average AUC = 0.85. Elevated IL-1β was associated with delivery within 14 days of assessment with AUC = 0.85 (0.67–0.96). Overall, elevated MMP-9 at GTP3 had the highest (13.3) positive likelihood ratio for distinguishing women at risk of sPTB. Lastly, a positive correlation between MMP-9 and TIMP-1 at all GTPs (*ρ* ≥ 0.61, p < 0.01) for women delivering at term was only observed at GTP1 for those who delivered preterm (*ρ* = 0.70, p < 0.03).

**Conclusions:**

In this cohort, sPTB is associated with gestation-dependent increase in pro-inflammatory cytokines, decreased IL-10 and GM-CSF, and dysregulated MMP-9-TIMP-1 interaction. Levels of cytokine (especially IL-1β) and ECM remodelling proteins rise significantly in the final 2 weeks before the onset of labour when sPTB is imminent. The signalling mechanisms for these ECM remodelling observations remain to be elucidated.

## Introduction

The burden of preterm birth (PTB, i.e., birth <37 completed weeks of gestation) is highest in low-resource settings such as Africa and Asia (1, 2) where the number of primary studies on the condition is lowest (3). In 2014, sub-Saharan African and Asian countries accounted for more than 80% of global PTBs, with rates in South Africa estimated at 12.4% (1). By 2020, southern Asia and sub-Saharan Africa accounted for approximately 65% of all PTBs globally (2). The PTB rate in sub-Saharan Africa has remained fairly unchanged (10.1%) between 2010 and 2020. However, the total number of preterm babies in this subregion increased by 563,100 between the same decade (2). PTB is a notable cause of neonatal mortality and morbidity (1, 4, 5) and accounted for approximately 18% of the 5.3 million deaths of children under 5 years that occurred in 2019 (4). Several modifiable risk factors including food insecurity, low socioeconomic status, smoking, and alcohol consumption during pregnancy are linked with poor birth outcomes including PTB (6, 7).

Intrauterine infection/inflammation (IUI) is responsible for >40% of PTBs (7–9). Inflammation-induced remodelling of the cervix and foetal membranes that underpins sPTB (10, 11) may vary by maternal demographics, environment (12), and vaginal microbiota (10, 13, 14). For example, in an Asian cohort sampled at the Thailand–Myanmar border, decreased interleukin (IL)-4, interferon gamma (IFN-γ), and tumour necrosis factor (TNF)-α were associated with vaginal microbiota deficient in *Lactobacillus crispatus* and *Finegoldia* but rich in *Prevotella buccalis* (15). In South African women, lactobacilli from a non-optimal microbiota (Nugent score 4–10) induced greater pro-inflammatory cytokine production than those from women with optimal microbiota (Nugent score 0–3) (16). In pregnant Caucasian women living with HIV infection, a suboptimal vaginal microbiota was associated with inflammation and release of matrix degrading enzymes—increased cervicovaginal fluid (CVF) MMP-9 which was related to elevated TIMP-1, IL-1β, IL-8, TNF-α, and polymorphonuclear leucocyte (17).

It has been hypothesised that ethnicity may influence PTB rates through gene-mediated variation in cytokine response to infection. African-Americans with a promoter polymorphism of the *MMP-9* gene produce increased MMP-9 that increases the risk for preterm premature rupture of membranes (PPROM) (18). The patho-mechanisms of cytokine-induced matrix degradation and sPTB in South African women without HIV infection are poorly understood. This study reports the relationships between markers of inflammation-driven remodelling of gestational tissues associated with sPTB in South African women. We describe the expression profiles of CVF cytokine and cervical tissue remodelling proteins MMP-9, and its regulator TIMP-1, in pregnant women at risk of PTB, and their associations with sPTB.

## Methods

This study was approved by the Health Research Authority (HRA) and Health and Care Research Wales (HCRW, 18/LO/2044); Research Ethics Committee of Faculty of Health Sciences, University of Pretoria (145/2019); and University of Cape Town, Faculty of Health Sciences Human Research Ethics Committee (UCT FHS HREC, 196/2019).

### Study participants

**Figure 1:**
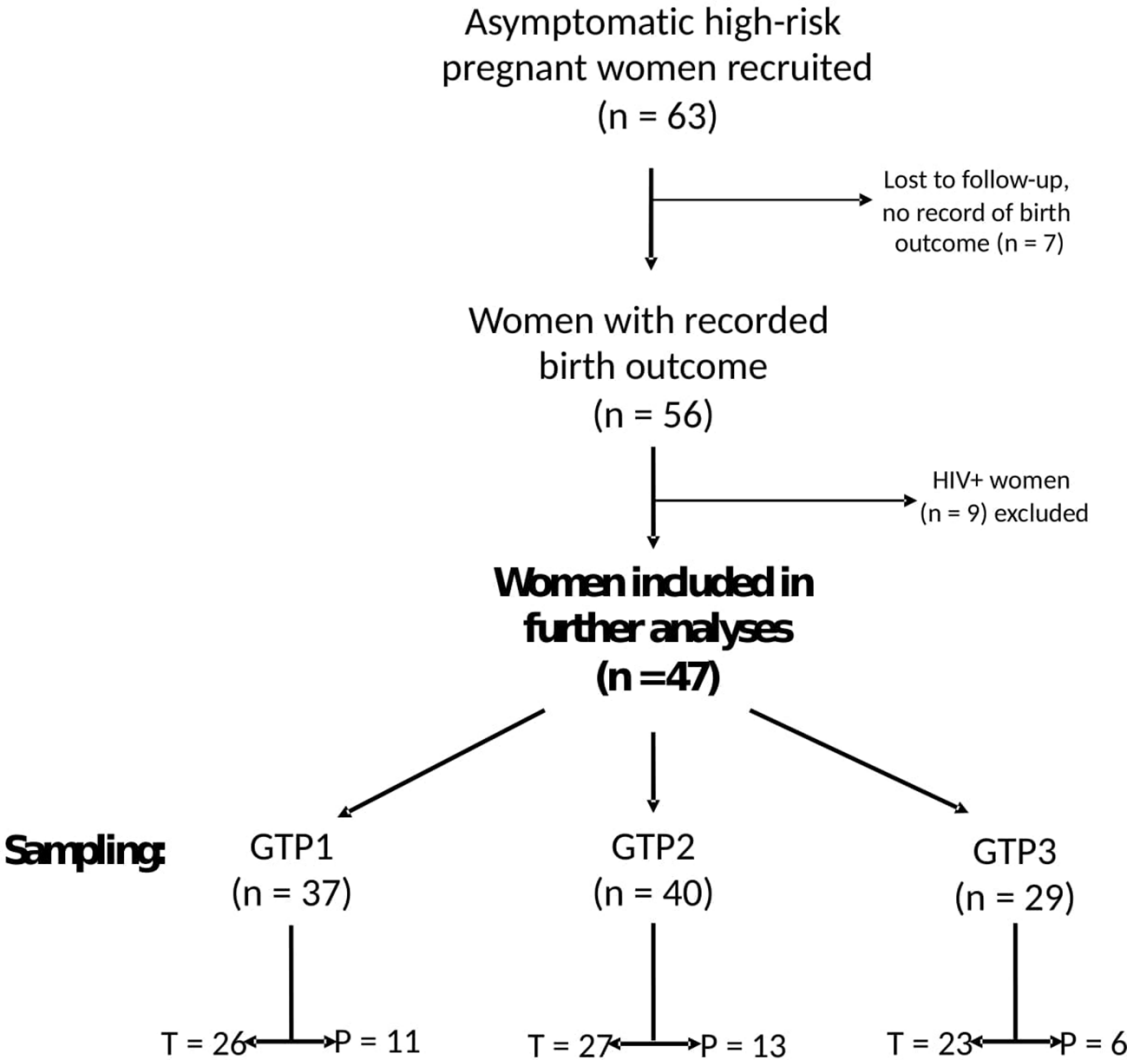
Flowchart of study participants and sampling. Only samples from 47 women whose birth outcomes were recorded and negative for any clinical genital tract infection were analysed for cytokines and extracellular matrix remodelling proteins. *GTP*, gestational time point; GTP1 (20–22 weeks), GTP2 (26–28 weeks), GTP3 (34–36 weeks). *T*, term; *P*, preterm.

A total of 63 participants were recruited from two study sites in South Africa, i.e., Groote Schuur Hospital, Cape Town (N = 47), and Steve Biko Academic Hospital, Pretoria (N = 16). Of these, seven (11.1%, Cape Town = 4 and Pretoria = 3) were lost to follow-up and their birth outcomes could not be ascertained. We therefore analysed data from 56 asymptomatic pregnant South African women at high risk of sPTB who presented for antenatal care and were sampled at three gestational time points (GTPs): GTP1 (20–22 weeks), GTP2 (26–28 weeks), and GTP3 (34–36 weeks) (**Figure 1**). These GTPs were informed by our previous observation of changes in CVF cytokines from 20–22 weeks to 26–28 weeks and up to 36 weeks that were associated with the risk of sPTB in a cohort of predominantly Caucasian women (19, 20). Furthermore, majority of sPTB occur around 32–<37 weeks (2, 9). The women considered in the current study were termed high risk due to previous history of PTB or mid-trimester pregnancy loss (MTL) between 14 and <37 weeks’ gestation (21–23). Other inclusion criteria were maternal age ≥18 years, singleton gestation, intact foetal membranes, and no known clinical urogenital tract infection. The participants included were not treated with vaginal progesterone or cervical cerclage. Women with multiple gestation, those who were uncertain of their gestation duration, and/or those who were on antibiotic medication were excluded. sPTB, defined as spontaneous delivery before 37 completed weeks of gestation, was the primary outcome of this study. Written informed consent was obtained from all participants before sample collection.

### CVF sample collection and preparation

At presentation, CVF samples were obtained from the posterior vaginal fornix of each pregnant woman with sterile Dacron swabs (Deltalab Eurotubo 300263) and stored immediately at −80°C. Following material transfer agreements, the samples were subsequently transferred on dry ice to our laboratory at the University of Sheffield, Sheffield, UK. Here, the swabs were processed by vortexing (washing) the cut end of the swab (containing the CVF sample) in a 500 μL solution of sterile methanol:water (50:50)% (v/v) for 5 min. Afterward, the swab was removed and stored at −80°C. The remaining CVF in solution was centrifuged for 3 min at 16,000 × *g* after which the supernatant was aspirated into a fresh tube and preserved at −80°C until further analysis. Part (100 μL) of the stored sample was used for metabolomics analysis by mass spectrometry (*unpublished data*), whereas 30 μL was employed for protein analysis by automated enzyme-linked immunosorbent assay (ELISA) as described below.

### Protein analysis by ELISA

The concentrations of 10 inflammatory cytokines—interleukin (IL)-1β, IL-4, IL-6, IL-8, IL-10, granulocyte-macrophage colony-stimulating factor (GM-CSF), monocyte chemoattractant protein 1 (MCP1, CCL2), monokine induced by interferon gamma (MIG or CXCL9), interferon gamma-induced protein 10 (IP-10 or CXCL10), RANTES (regulated on activation, normal T cell expressed and secreted)—and collagenase matrix-metalloproteinase-9 (MMP-9) and its regulator tissue inhibitor of metalloproteinase-1 (TIMP-1) were determined in the CVF samples by automated ELISA (Ella™ Automated Immunoassay System, ProteinSimple, Bio-Techne, USA). Three Simple Plex™ Cartridge Kits (ProteinSimple, Bio-Techne, USA) for 32 samples, for use with Human Plasma/Serum—three analytes IL-8, RANTES, and TIMP-1 (SPCKC-PS-008095); eight analytes CCL2, CXCL10, GM-CSF, IL-10, IL-1β, IL-4, IL-6, MIG (SPCKC-PS-007928); and MMP-9 (SPCKC-PS-000661)—were used according to the manufacturer’s instructions. These cartridge kits were supplied with sample diluents (SD13) which were used to dilute the CVF samples (30:30 μL, 1:2 dilution), and 50 μL of the diluted samples was added to the sample wells on the cartridge. Similarly, 50 μL each of reconstituted high-quality (HQC) and low-quality (LQC) controls (1:1 dilution) was added to the last two sample wells on the cartridge. The standards (controls) for each of the 12 analytes of interest were reconstituted according to the certificate of analysis (CoA) of the manufacturer (ProteinSimple, Bio-Techne, USA). After addition of 1 mL of Wash Buffer A to all buffer inlets and peeling the protective lining from the bottom of the cartridge, the cartridge was placed into the Ella’s cartridge holder and clamped securely. The samples were run on an Ella-20090625 analyser using the Simple Plex Runner application (version 3.8.0.12, Bio-Techne, USA) and analysed by the Simple Plex Explorer (version 3.8.0.12, Bio-Techne, USA). Final concentrations of the analytes were calculated from the mean signal using analyte/lot-specific factory calibration curves (RFU vs. concentration (pg/mL) produced at ProteinSimple (Bio-Techne, USA) on a lot-to-lot basis for the assay.

### Statistical analyses

The protein concentrations (pg/mL) were log-transformed to attain approximate normality. The distribution of continuous data was confirmed by Shapiro–Wilk and Anderson–Darling tests. The continuous clinical and demographic data of the participants were presented as median and interquartile range (IQR) and compared between term- and preterm-delivered women using the Mann–Whitney *U*-test (except stated otherwise). The log-transformed protein concentration values were compared between the groups by Welch’s t-test and Mann–Whitney (compare ranks) tests when comparing two groups or Welch’s ANOVA or Kruskal–Wallis tests for more than two independent groups. Categorical variables and frequencies were compared between the groups by Chi-squared and Fisher’s exact tests. Correlation between proteins was determined by Spearman’s correlation coefficient (*ρ, rho*). Furthermore, we employed the area under the receiver operating characteristic (AUC) curve to determine the predictive capacity of the proteins for subsequent sPTB. Probability (*p*) values < 0.05 were considered statistically significant. All data were analysed using GraphPad Prism 10.0.2 (232) (GraphPad Software, Inc. CA, USA), and MedCalc v22.014 (MedCalc Software Ltd, Ostend, BE; http://www.medcalc.org; 2023) statistical software packages.

## Results

### Characteristics of study participants

A total of 56 (out of the 63 recruited) singleton pregnant women at high risk of PTB completed the study, and their birth outcomes were recorded (**Figure 1**). The participants’ demographic and clinical details, including birth outcomes, are presented in **Table 1**. Maternal age and body mass index (BMI) did not significantly differ between women who delivered term compared with preterm. Majority (94.6%) of the participants were of Black and mixed-race ethnicities. As expected, the preterm babies had low birth weight (LBW, <2,500 g) and weighed less (p<0.0001) than their term counterparts (**Table 1**). Most of the women who delivered preterm babies delivered between 32 and 36 weeks of gestation (GTP1 = 82%, GTP2 = 92%, GTP3 = 100%). Nine study participants (Cape Town = 7 and Pretoria = 2) were subsequently found to be women with HIV, and their samples were excluded from further analyses. Hence, only samples from 47 participants were analysed for cytokines and ECM remodelling proteins (**Figure 1**). The demographic and clinical details, including birth outcomes of this subset of participants (n = 47: Cape Town = 36 and Pretoria = 11), are presented in **Table 2**.

**Table 1:** Participants’ clinical and demographic details according to birth outcome.

Clinical/demographic factor	Term (n=36)	Preterm (n=20)	*p* value
Age (years)	32.3 ± 5.2 (n=36)	29.3 ± 5.2 (n=20)	**0.04**
BMI (kg/m^2^)^a^	31.5 ± 7.4 (n=28)	28.2 ± 5.5 (n=15)	0.10
GAAD (weeks)	38 (38-39) (n=36)	34 (29.5-36.0) (n=20)	**<0.0001**
Birth weight (g)	3,202 (2,940–3,483) (n=36)	2,330 (1,398–2,669) (n=20)	**<0.0001**
Race/ethnicity (n, %)	Black = 20 (55.6) White = 0 (0) Mixed = 16 (44.4)	Black = 8 (40) White = 3 (15) Mixed = 9 (45)	**0.049**
Smoker (n, %)	Yes = 1 (2.8) No = 35 (97.2)	Yes = 2 (10) No = 18 (90)	0.26
Previous history of PTB/MTL (n, %)	Yes = 36 (100) No = 0	Yes = 19 (95) No = 1 (5)	0.36
Spontaneous labour (n, %)	Yes = 15 (41.7) No = 20 (55.6) NR = 1 (2.8)	Yes = 16 (80) No = 3 (15) NR = 1 (5)	**0.004**

Maternal age and body mass index (BMI) are presented as mean ± standard deviation and compared between the groups by Welch’s t test, whereas gestational age at delivery (GAAD) and birth weight data are presented as median with interquartile range (IQR) and compared between the groups by Mann–Whitney U-test. Data on race/ethnicity, smoking, and previous history of PTB/MTL and spontaneous labour were compared between the groups by Chi-squared and Fisher’s exact tests. MTL, mid-trimester pregnancy loss; NR, data not recorded; PTB, preterm birth. ^a^Low n numbers = data not recorded/unknown.

The significant p values are highlighted in bold.

**Table 2:** Clinical and demographic details of participants whose cervicovaginal fluid samples were analysed for cytokines and extracellular matrix remodelling proteins.

Clinical/demographic factor	Term (n=32)	Preterm (n=15)	*p* value
Age (years)	32.3 ± 5.5 (n=32)	28.9 ± 5.9 (n=15)	0.065
BMI (kg/m^2^)^a^	31.4 ± 7.6 (n=24)	27.9 ± 5.1 (n=10)	0.13
GAAD (weeks)	38 (38.0–39.0) (n=32)	35 (32.0–36.0) (n=15)	**<0.0001**
Birth weight (g)	3,202 (3,015–3,483) (n=32)	2,380 (1,980–2,700) (n=15)	**<0.0001**
Race/ethnicity (n, %)	Black = 16 (50) White = 0 (0) Mixed = 16 (50)	Black = 4 (26.7) White = 3 (20) Mixed = 8 (53.3)	**0.02**
Smoker (n, %)	Yes = 1 (3.1) No = 31 (96.9)	Yes = 2 (13.3) No = 13 (86.7)	0.24
Previous history of PTB/MTL (n, %)	Yes = 32 (100) No = 0	Yes = 13 (86.7) No = 2 (13.3)	0.10
Spontaneous labour (n, %)	Yes = 14 (43.8) No = 17 (53.1) NR = 1 (3.1)	Yes = 12 (80) No = 2 (13.3) NR = 1 (6.7)	**0.02**

Maternal age and body mass index (BMI) are presented as mean ± standard deviation and compared between the groups by Welch’s t test, whereas gestational age at delivery (GAAD) and birth weight data are presented as median with interquartile range (IQR) and compared between the groups by Mann–Whitney U-test. Data on race/ethnicity, smoking, and previous history of PTB/MTL and spontaneous labour were compared between the groups by Chi-squared and Fisher’s exact tests. MTL, mid-trimester pregnancy loss; NR, data not recorded; PTB, preterm birth. ^a^Low n numbers = data not recorded/unknown.

The significant p values are highlighted in bold.

### Cross-sectional analyses of CVF cytokines, MMP-9, and TIMP-1 in the second and third trimesters by birth outcome

The concentrations of 10 inflammatory cytokines and MMP-9 and TIMP-1 in CVF samples were determined by ELISA at three GTPs: GTP1 (20–22 weeks, n = 37), GTP2 (26–28 weeks, n = 40), and GTP3 (34–36 weeks, n = 29). Of these samples, 11 (GTP1), 13 (GTP2), and 6 (GTP3) were from women who subsequently delivered preterm (**Figure 1**). The women who delivered preterm did so within 85.3 ± 25.9, 51.3 ± 15.3, and 11.8 ± 7.5 (mean ± SD) days after sampling at 20–22 weeks (GTP1), 26–28 weeks (GTP2), and 34–36 weeks (GTP3), respectively.

**Figure 2:**
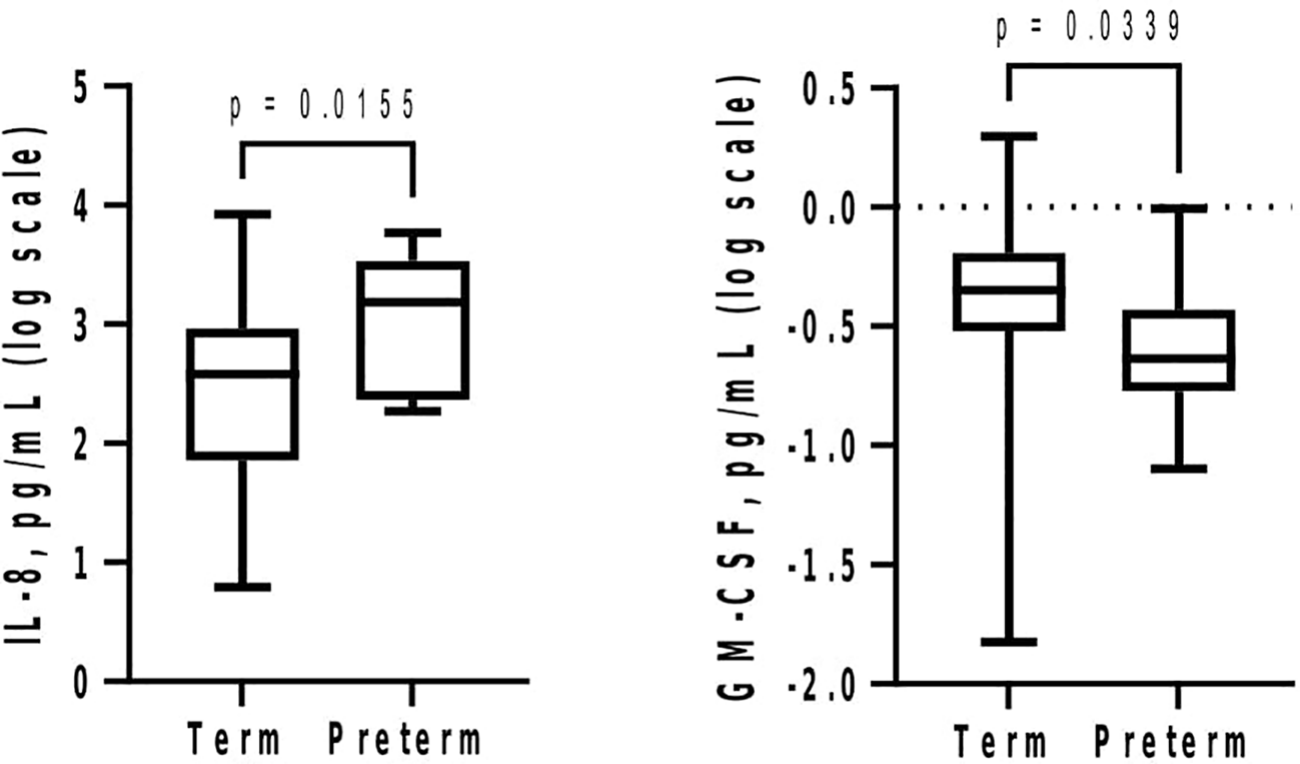
Mid-second trimester (20–22 weeks) increase in cervicovaginal fluid interleukin-8 (IL-8) and decrease in granulocyte-macrophage colony-stimulating factor (GM-CSF) are associated with spontaneous preterm birth. Protein concentrations (pg/mL) are presented as logarithm scale. Welch’s t test: IL-8 (term = 26, preterm = 11); Mann–Whitney *U*-test: GM-CSF (term = 22, preterm = 10).

**Figure 3:**
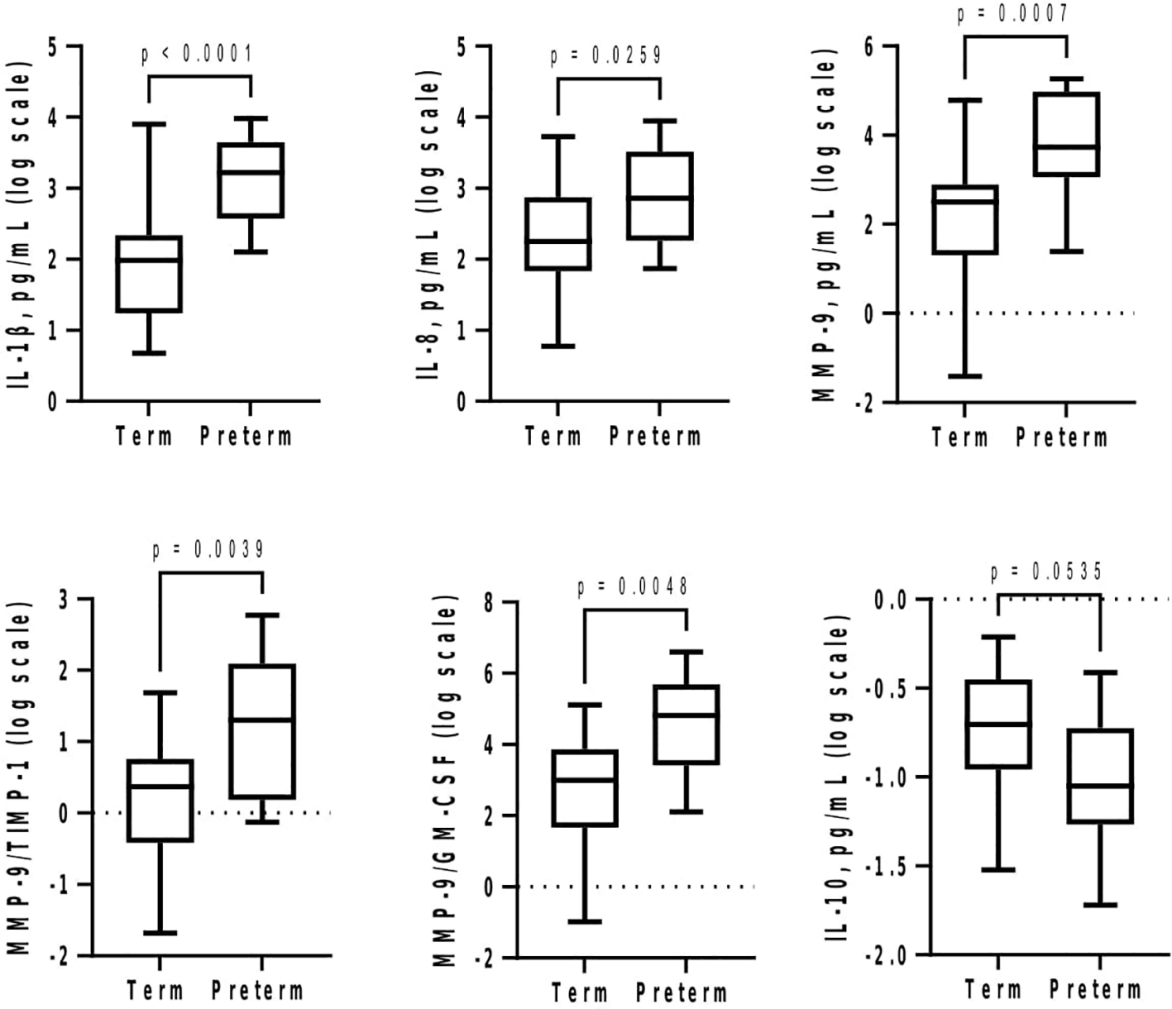
Cervicovaginal fluid pro-inflammatory cytokines and extracellular matrix remodelling proteins increase at late second trimester (26–28 weeks) in women who deliver preterm, whereas the anti-inflammatory IL-10 tend to increase in women who deliver at term. Protein concentrations (pg/mL) are presented as logarithm scale. Welch’s t test: IL-8, MMP-9, MMP-9/TIMP-1 ratio (term = 27, preterm = 13); IL-1β (term = 27, preterm = 12); MMP-9/GM-CSF ratio (term = 25, preterm = 10). Mann–Whitney *U*-test: IL-10 (term = 20, preterm = 7). *GM-CSF*, granulocyte-macrophage colony-stimulating factor; *IL*, interleukin; *MMP-9*, matrix-metalloproteinase-9; *TIMP-1*, tissue inhibitor of metalloproteinase-1.

At GTP1, IL-8 (4-fold, p = 0.02) was higher whereas GM-CSF (~1.4-fold, p = 0.03) was lower in the women who subsequently delivered preterm compared with term-delivering women (**Table 3** and **Figure 2**). However, at GTP2, IL-1β (~18-fold, p < 0.0001), IL-8 (4-fold, p = 0.03), MMP-9 (>17-fold, p = 0.0007), MMP-9/TIMP-1 ratio (>8-fold, p = 0.004), and MMP-9/GM-CSF ratio (~87-fold, p = 0.005) were higher in preterm—compared with term-delivering women. Logistic regression also showed an association between increased IL-1β and risk of sPTB (OR = 14.3, 95% CI = 1.07–190.25). By contrast, there was a trend towards higher IL-10 (4-fold, p = 0.05) in the term compared with the preterm women (**Table 3** and **Figure 3**).

**Table 3:** Predictive capacity of cervicovaginal proteins for spontaneous preterm birth and delivery within 14 days of index assessment at 34–36 weeks.

Protein (pg/mL)	AUC (95% CI)	Sens (%)	Spec (%)	PPV (%)	NPV (%)	PLR	Cut-off (pg/ml)
Spontaneous preterm birth							
**GTP1 (20–22 weeks)**							
IL-8	0.71 (0.54–0.85)	100	42.3	42.3	100	1.73	>107
GM-CSF	0.74 (0.55–0.88)	90	68.2	56.2	93.7	2.83	≤0.38
**GTP2 (26-28 weeks)**							
IL-1β	0.89 (0.74–0.97)	91.7	77.8	64.7	95.5	4.1	>219
IL-8	0.71 (0.55–0.84)	76.9	66.7	52.6	85.7	2.3	>299
IL-10	0.75 (0.55–0.90)	71.4	85	62.5	89.5	4.8	≤0.098
MMP-9	0.84 (0.69–0.94)	84.6	74.1	61.1	90.9	3.3	>634
MMP-9/TIMP-1 ratio	0.76 (0.60–0.88)	69.2	81.5	64.3	84.6	3.7	>5.9316
MMP-9/GM-CSF ratio	0.80 (0.64–0.92)	70	84	63.6	87.5	4.4	>16955.2
**GTP3 (34-36 weeks)**							
IL-1β	0.94 (0.78–0.99)	100	82.6	60	100	5.8	>393
IL-8	0.86 (0.68–0.96)	83.3	78.3	50	94.7	3.8	>832
MMP-9	0.80 (0.60–0.93)	66.7	95	80	90.5	13.3	>36443
TIMP-1	0.79 (0.59–0.92)	83.3	71.4	45.5	93.8	2.9	>1123
Delivery ≤ 14 days of assessment at GTP3							
IL-1β	0.85 (0.67-0.96)	100	76	40	100	4.2	>393

Protein concentrations (pg/mL) were log-transformed. GTP1 (term = 26, preterm = 11); GTP2 (term = 27, preterm = 13); and GTP3 (term = 23, preterm = 6, delivery ≤14 days of assessment = 4, delivery >14 days of assessment = 25). Cut-off (or criterion), concentration that indicates the likelihood of spontaneous preterm birth. IL, interleukin; MMP-9, matrix-metalloproteinase-9; TIMP-1, tissue inhibitor of metalloproteinase-1; GTP, gestational time point; AUC, area under the ROC curve; CI, confidence interval; Sens, sensitivity; Spec, specificity; p value, probability (significance); PPV, positive predictive value; NPV, negative predictive value; PLR, positive likelihood ratio.

**Figure 4:**
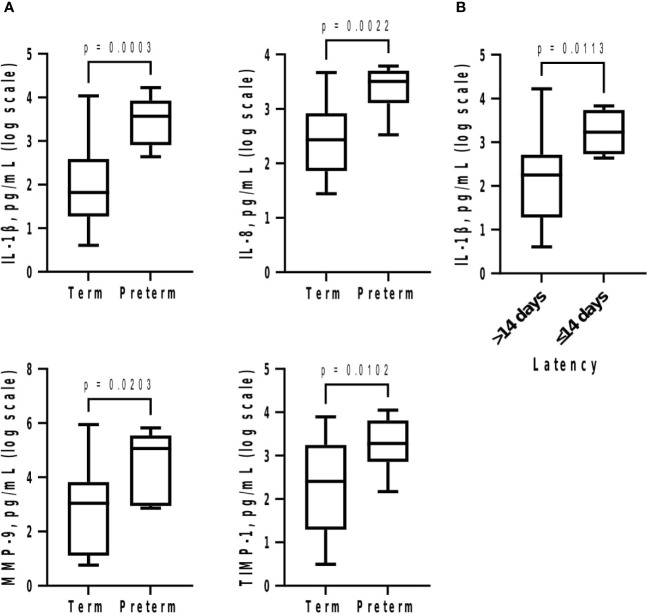
**(A)** Cervicovaginal fluid pro-inflammatory cytokines and extracellular matrix remodelling proteins are increased at 34–36 weeks in women who deliver preterm; and **(B)** delivery within 2 weeks of index assessment is associated with increased IL-1β. Protein concentrations (pg/mL) are presented as logarithm scale. Welch’s t test: IL-1β, IL-8 (term = 23, preterm = 6); MMP-9 (term = 21, preterm = 6); TIMP-1 (term = 21, preterm = 6); delivery within 2 weeks for IL-1β (>14 days = 25, ≤14 days = 4). *IL*, interleukin; *MMP-9*, matrix-metalloproteinase-9; *TIMP-1*, tissue inhibitor of metalloproteinase-1.

Subsequently at GTP3, IL-1β (>58-fold, p = 0.0003), IL-8 (~12-fold, p = 0.002), MMP-9 (~296-fold, p = 0.02), and TIMP-1 (~35-fold, p = 0.01) were higher in preterm compared with term women (**Table 3**; **Figure 4A**). Logistic regression also showed an association between increased IL-1β and risk of sPTB (OR = 8.2, 95% CI = 1.02-66.55). Furthermore, increased IL-1β was observed in the women who delivered preterm within 2 weeks of index assessment (p = 0.01) (**Figure 4B**).

### Longitudinal analyses of CVF cytokines, MMP-9, and TIMP-1 from the second to third trimesters by birth outcome

**Figure 5:**
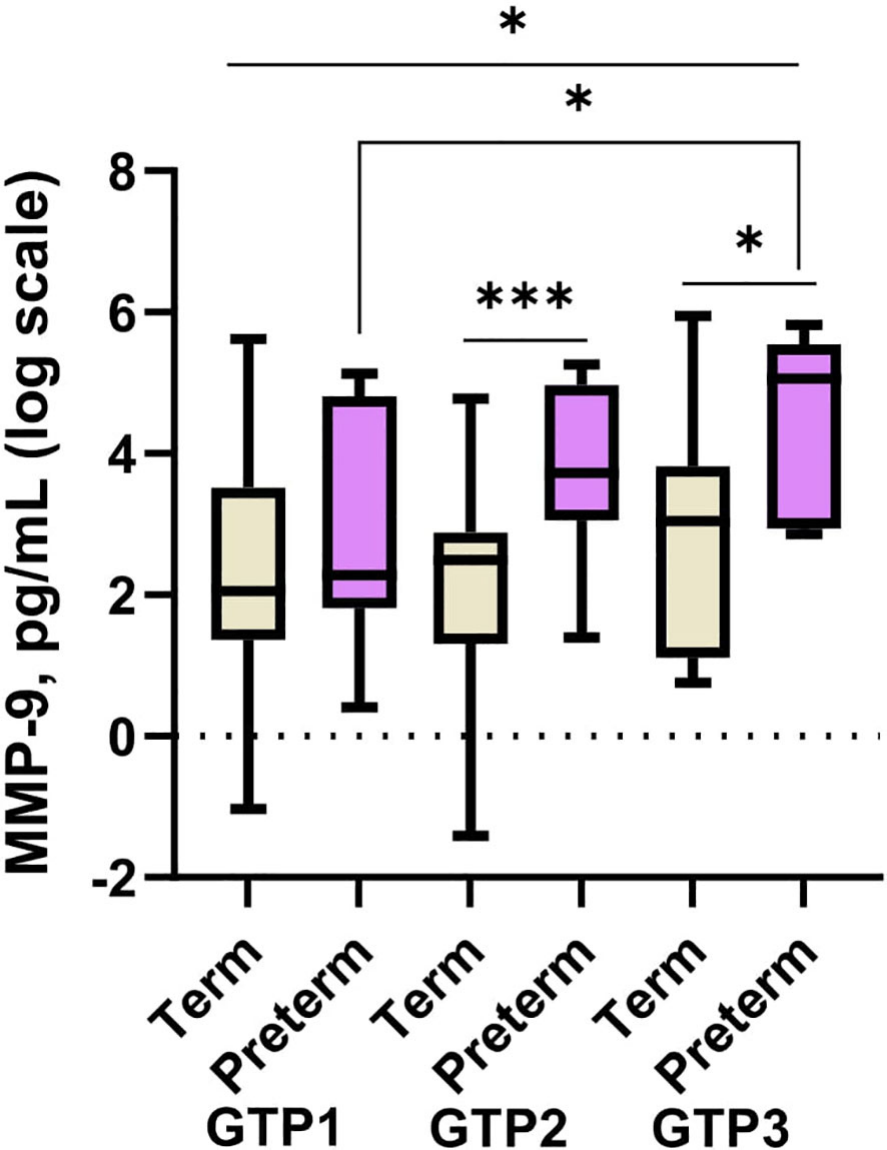
Cervicovaginal fluid matrix-metalloproteinase-9 (MMP-9) increase from mid-second to third trimester in women who deliver preterm. MMP-9 is higher in women who deliver preterm than those that deliver at term at late second and third trimesters. Protein concentrations (pg/mL) are presented as logarithm scale. Welch’s t test: for two groups and Welch’s ANOVA test: more than two groups. GTP1 (20–22 weeks), GTP2 (26–28 weeks), and GTP3 (34–36 weeks). *GTP*, gestational time point. *p < 0.05, ***p = 0.0007.

Between GTP1 and GTP3, only MMP-9 increased (p = 0.045) in the preterm women (**Figure 5**). None of the other proteins changed significantly from one GTP to the other according to birth outcome.

Overall (term and preterm women combined), there was a positive correlation between MMP-9 and TIMP-1 at the three GTPs, which was maintained in the term-delivered women (GTP1 *ρ* = 0.80, p < 0.0001; GTP2 *ρ* = 0.74, p < 0.0001; and GTP3 *ρ* = 0.61, p = 0.005) but retained in the preterm-delivered women only at GTP1 (*ρ* = 0.70, p = 0.03) when subanalysed separately (**Supplementary Table S1**, **S2**; and **Supplementary Figure S1**). However, the MMP-9/TIMP-1 ratio was higher in the preterm compared with the term women at GTP2 only (**Figure 3**).

Moreover, there were correlations between the other inflammatory mediators at the three GTPs in each group of women and when both groups were combined (**Supplementary Figure S1** and **Supplementary Tables S1**, **S2**). Notably, at the three GTPs, IL-1β correlated positively with MMP-9 and TIMP-1 in the term-delivered women.

IL-1β also correlated positively with MMP-9 and TIMP-1 in the preterm women at GTP1 and GTP2 only. The preterm women also showed negative correlation between GM-CSF and CCL2 at GTP1 only (**Supplementary Figure S1** and **Supplementary Table S1**).

### Receiver operating characteristic curve analysis

Elevated IL-8 (AUC, 95% CI: 0.71, 0.54–0.85) and GM-CSF (0.74, 0.55–0.88) at GTP1 as well as IL-1β (0.89, 0.74–0.97), IL-8 (0.71, 0.55–0.84), MMP-9 (0.84, 0.69–0.94), MMP-9/TIMP-1 ratio (0.76, 0.60–0.88), and MMP-9/GM-CSF ratio (0.80, 0.64-0.92) at GTP-2 were associated with sPTB. By contrast, decreased IL-10 (0.75, 0.55-0.90) was associated with sPTB at GTP2 only (**Table 3**).

**Figure 6:**
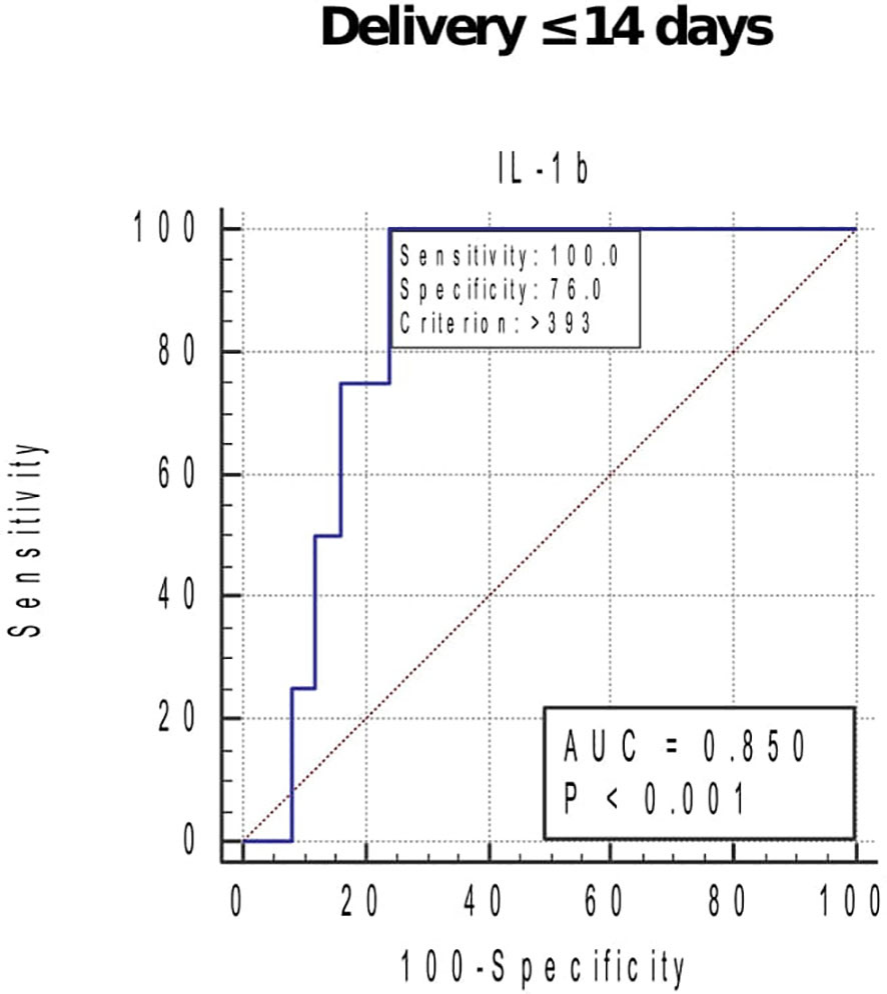
Receiver operating characteristic curve (ROC) analysis of the cervicovaginal fluid interleukin (IL)-1β for delivery within 14 days of index assessment at 34–36 weeks. Criterion (cut-off), concentration that indicates the highest likelihood of delivery in approximately 2 weeks of assessment. *AUC*, area under the ROC curve. Only one woman sampled at 26–28 weeks, and none at 20–22 weeks delivered within 2 weeks of index assessment.

At GTP3, elevated IL-1β (0.94, 0.78–0.99), IL-8 (0.86, 0.68–0.96), MMP-9 (0.80, 0.60–0.93), and TIMP-1 (0.79, 0.59–0.92) were associated with sPTB. These biomarkers had sensitivities, specificities, and positive and negative predictive values ranging from 42.3% to 100% (**Table 3**). Overall, elevated MMP-9 at GTP3 had the highest (13.3) positive likelihood ratio for distinguishing women at risk of sPTB from those who would eventually deliver at term. Furthermore, elevated IL-1β at GTP3 was associated with delivery within 14 days of assessment (0.85, 0.67–0.96) (**Table 3** and **Figure 6**). Only two women delivered within 7 days of index assessment.

## Discussion

We studied the temporal changes in cervicovaginal cytokines and ECM remodelling proteins in a predominantly Black and mixed-race South African cohort in order to better understand the inflammatory responses/profiles that drive the pathway to sPTB in this region/context, which could guide biomarker discovery and formulation of preventive and therapeutic interventions. This study demonstrates that in vaginal swab samples from non-labouring South African women at high risk of sPTB obtained at mid- to late-second and third trimesters, elevated IL-8 at 20–22 weeks (GTP1); IL-1β, IL-8, MMP-9, MMP-9/TIMP-1 ratio, and MMP-9/GM-CSF ratio at 26–28 weeks (GTP2); and IL-1β, IL-8, MMP-9, and TIMP-1 at 34–36 weeks (GTP3) were associated with sPTB, whereas elevated GM-CSF at 20–22 weeks and IL-10 at 26–28 weeks of gestation were associated with delivery at term. Secondly, increased IL-1β assessed at 34–36 weeks was associated with delivery within 2 weeks of index assessment. Thirdly, between 20–22 and 34–36 weeks, MMP-9 increased significantly with associated increase in IL-1β in the preterm women but not in the term-delivered women. The increased MMP-9 particularly at GTP3 indicated a large and significant increase in the probability of sPTB. Furthermore, we observed that the positive (regulatory) correlation between MMP-9 and TIMP-1 in term-delivered women was absent in the preterm women at later gestation (GTP 2 and GTP3) near the onset of labour. Moreover, moderate to late PTB subtypes (i.e., delivery between 32 and <37 weeks) (2, 9) predominated the preterm group.

Increased IL-1β (the master cytokine in inflammation) (24–26) was related to increased MMP-9 and TIMP-1 in the term women at all GTPs. Conversely, increase in IL-10, an anti-inflammatory cytokine (25, 27), was associated with term delivery at GTP2. The positive relationship between IL-1β, MMP-9, and TIMP-1 was also observed in the preterm women but only at GTP1 and GTP2. The breakdown of the positive (regulatory) relationship between MMP-9 and TIMP-1 in the preterm women at later gestation (particularly at GTP3) may be one of the driving mechanisms of sPTB in this cohort. Overexpression of MMP-9 in gestational tissues without a commensurate increase in its inhibitor (TIMP-1) creates dysregulated extracellular matrix (ECM) remodelling that induces premature cervical ripening, PROM, and PTB (10, 28).

Our observation in this South African cohort differs from our previous study of a UK population of similarly asymptomatic high-risk women but predominantly of Caucasian ancestry, which showed no difference in individual CVF cytokine levels between term vs. preterm at 20–22 and 26–28 weeks (19). That is, while the South African women who experienced sPTB had higher concentrations of inflammatory cytokines compared with their term counterparts at 20–22, 26–28, and 34–36 weeks, the preterm-delivered women in the previous UK Caucasian cohort did not show such differences at 20–22 and 26–28 weeks’ gestation, although they were not studied at 34–36 weeks. Only an increase in RANTES and IL-1β from 20 + ^0^–22 + ^6^ to 26 + ^0^–28 + ^6^ weeks expressed as a ratio was higher in preterm vs. term women in the UK cohort (19). Additionally, CVF cytokine concentrations were generally higher at mid-pregnancy in the UK cohort than the South African women. These context-specific variations between South African (majorly Black African and mixed race) versus UK (mainly Caucasian) cohort is consistent with our hypothesis that the mechanism of inflammation-induced sPTB may vary by race and geographical location.

It is also noteworthy that in the current study, IL-6 did not differ significantly between the term vs. preterm women at any GTP. This is despite a recent report of the ability of IL-6 to predict the onset of labour regardless of gestational age at sampling (29). The disparity between the reports is not surprising because while we studied a cohort of predominantly Black African and mixed (White/Black) non-labouring women, the other study (29) reported that IL-6 was predictive of onset of labour in Mexican women in the active phase of labour (regular uterine contractions with cervical dilation >3 cm). However, this report is similar to our previous observation that elevated CVF IL-6 is predictive of sPTB in a cohort of women with established labour, albeit of Caucasian descent (20). This may imply that CVF IL-6 is a major mediator of the inflammatory responses leading to the premature onset of labour in White and Hispanic labouring women, but not implicated in the pathway to spontaneous labour in Black African or women African decent without symptoms of spontaneous preterm labour (PTL). Again, this supports our hypothesis that the inflammatory responses that trigger the onset of PTL are influenced by racioethnic and environmental factors.

Various studies have reported the role of genetic polymorphisms in the regulation of cervical ECM remodelling. Apart from African-Americans with PPROM-associated *MMP-9* gene polymorphisms (18), Indian women with the *IL-6* G allele express increased MMP-9 and sPTB (30). However, a definite association between *TLR-4, IL-1α, IL-1β, IL-6*, and *IL-10* gene polymorphisms and PTB in diverse ethnic and environmental settings has not been demonstrated (12). Further studies of the impact of gene–environmental interactions on sPTB (31, 32) may clarify these contextual differences in PTB incidence and patterns. Moreover, the inflammatory responses to infectious and non-infectious stimuli (environmental exposure) may be altered by inherent genetic susceptibility mediated by such gene polymorphisms (31, 32).

Herein, we hypothesise that the proteolytic activity of MMP-9 may be elaborated by IL-1β above the anti-proteolytic activity of TIMP-1 in women destined to deliver prematurely. The cytokine inhibitory action of IL-10 may also be diminished especially at the second to third trimester when sPTB is imminent. Conversely, there appears to be a balanced MMP-9/TIMP-1 interplay in the term women as both proteins increased in tandem across all GTPs. This balanced interaction is defective (absent) in the preterm women who demonstrated higher levels of IL-1β, MMP-9, TIMP-1, and MMP-9/TIMP-1 ratio (at GTP2 and GTP3); a positive relationship between all three proteins at GTP1 and GTP2 only; and a decrease in the anti-inflammatory IL-10 (25, 27) at GTP2. The absence of a positive correlation between IL-1β and the regulatory TIMP-1 in the preterm women at GTP3 could possibly contribute to the premature stimulation of labour pathways.

Because we excluded women with clinical genital tract infection, and most of the preterm women experienced moderate to late sPTB (32 to <37 weeks) (2, 9), we hypothesise that the inflammation-associated sPTB in this cohort is more likely of the non-infectious nature. This is because earlier PTB (<30 weeks) is commonly triggered by lower reproductive tract infection ascending into the intrauterine/intraamniotic cavity (9, 33). That is, the lower the gestational age at presentation with spontaneous PTL (with intact or ruptured membranes), the higher the frequency of abnormal genital tract microbiota and intrauterine/intraamniotic infection (34–38). Infection and infection-driven inflammation are more prevalent (up to 79% in some studies) in extreme sPTB (<28 weeks) (39, 40).

Other acute pro-inflammatory cytokines such as IL-8 (25, 41), which was also elevated, may act synergistically with or independent of IL-1β to perpetuate this inflammatory sPTB drive regardless of the stimulus. Our observations are similar to other studies (42–45) that have also reported upregulation of this mediator in CVF, amniotic fluid, cervix, chorioamnion, and placenta of preterm-delivered women, and supported by strong predictive accuracy (>70% and predictive values between 42% and 100%) for imminent sPTB.

The predictive potentials of the markers for sPTB and delivery within 2 weeks of assessment at 34–36 weeks observed in this study appear comparable with clinically employed tests such as transvaginal ultrasound cervical length measurement and quantitative cervicovaginal foetal fibronectin (20, 46, 47), which are currently employed in high-resource settings, but not in settings with limited resources such as sub-Saharan Africa. Additional studies investigating whether these biomarkers (especially IL-1β) may predict delivery within 2 weeks of assessment of women presenting with symptoms of PTL to inform clinical care (48, 49) are required.

The patho-mechanisms of MMP/TIMP-induced ECM modulation during pregnancy and parturition have been extensively reported (10, 17, 50). Labour-associated inflammatory processes are mediated by the overexpression of IL-1β, IL-6, IL-8, MCP-1, RANTES, and TNF-α in gestational tissues including foetal membranes, cervix, amniotic fluid, CVF, and placenta (7, 19, 20, 25, 30, 51–53). Expression of pro-inflammatory mediators in turn causes (a) increased elaboration of prostaglandins (PG) and contraction-associated proteins, which promote uterine contractility; (b) degradation of the chorioamnion ECM; and (c) remodelling of the cervix by MMP-9 linked to decreased expression of TIMPs (7, 10, 51, 54–56).

Overproduction of IL-1β (as we observed) characterises labour with or without infection (57, 58) by inducing the labour pathway (57), especially in IUI-associated PTB (25). IL-1β increases production of MMPs such as MMP-9 in gestational tissues by increasing production of cyclooxygenase-2 and PGE_2_, vascular permeability, and immunocyte infiltration (51, 59–62). IL-1β can increase the production/activity of MMP-9 independently (63, 64) or in synergy with TNF-α (65), suggesting a causal relationship between IL-1β and IUI-associated PTB (51)—both intra-amniotic (64, 66–68) and systemic (69) infusion of IL-1β induced PTL and PTB.

On the contrary, IL-10 obliterates infectious induction of MMP-2 and -9 in chorioamnion (70), and cytotrophoblast MMP-9 activity/invasiveness in an autocrine fashion (71). IL-10 decreases MMP-9 activity and increases TIMP-1 levels in coculture systems in a dose-dependent manner (72). IL-10 reduces intra-amniotic concentrations of pro-inflammatory mediators and incidence of prematurity in animals challenged with lipopolysaccharide, *Escherichia coli*, and IL-1β (67, 73, 74). In line with these anti-inflammatory roles, we observed a decrease in IL-10 in term-delivered women at GTP2, which distinguished them from the preterm-delivered women with high sensitivity, specificity, PPV, NPV of >62%, and a +LR of 4.8. The role of IL-10 in regulating labour-associated inflammatory responses at different GTPs requires more race/environment-specific investigations.

Additionally, an imbalance in M1/M2 macrophage activation/polarization, which determines the fate of tissues in inflammation or injury (75–77), can plausibly be deduced from our data. The decrease in GM-CSF, an activator of M1 macrophages (75, 78), in the term women at GTP1 could be associated with decreased or regulated inflammation in these women. GM-CSF can induce the expression of IL-10 at the human maternal–foetal interface (79). Moreover, women who deliver at term showed a similar but early- (6–12 weeks) to mid-gestation (24 weeks) decline in plasma GM-CSF (80). Although the gestation-associated changes in GM-CSF were not significant in the preterm group, a decrease in GM-CSF correlated with an increase in CCL2, an activator of M2 macrophages (78) at GTP1. However, this relationship was absent at GTP3. Furthermore, CVF concentrations of both cytokines increase in women with a short cervix (81). Nonetheless, the role of M1/M2 macrophage polarisation in sPTB mediated by GM-CSF-CCL2 interaction is currently unclear.

Our study may be the first to investigate longitudinal changes in inflammatory mediators and matrix remodelling proteins in relation to sPTB in an African cohort of non-labouring women at risk of PTB. Although our findings are intriguing, interpretation should be with caution due to the relatively small and limited sample size. For instance, due to the limited number of women who provided samples at all three GTPs, there were no significant differences in the proteins when we performed a pairwise comparison between GTP1 and GTP2, GTP2 and GTP3, or GTP1 and GTP3 for either term or preterm women. Future studies are required to explore such comparisons as well as our previous observations that women presenting with symptoms of PTL who delivered within 2 weeks of index assessment demonstrated elevated RANTES, IL-6, and TNFR1 (20). Additionally, a linear mixed-effects model of pro-inflammatory cytokines and ECM remodelling proteins was not performed as the dependent variable (outcome) in this study was binary (sPTB or term) instead of continuous.

## Conclusions

In this cohort, sPTB is associated with a gestation-dependent increase in acute and chronic pro-inflammatory cytokines, a decrease in IL-10 and GM-CSF, and dysregulated MMP-9-TIMP-1 interaction. Levels of cytokine (especially IL-1β) and ECM remodelling proteins rise significantly in the final 2 weeks before the onset of labour when sPTB is imminent. Whether this imbalance is modulated by IL-1β and perhaps IL-10 and contributes to the extracellular tissue remodelling and collagen degradation that precede birth remains to be determined.

## Data availability statement

The original contributions presented in the study are included in the article/**Supplementary Material**. Further inquiries can be directed to the corresponding author.

## Ethics statement

The studies involving humans were approved by Health Research Authority (HRA) and Health and Care Research Wales (HCRW, 18/LO/2044); Research Ethics Committee of Faculty of Health Sciences, University of Pretoria (145/2019); and University of Cape Town, Faculty of Health Sciences Human Research Ethics Committee (UCT FHS HREC, 196/2019). The studies were conducted in accordance with the local legislation and institutional requirements. The participants provided their written informed consent to participate in this study.

## Author contributions

EA: Conceptualization, Data curation, Formal analysis, Funding acquisition, Investigation, Methodology, Project administration, Resources, Software, Supervision, Validation, Visualization, Writing – original draft, Writing – review & editing. NI: Data curation, Investigation, Methodology, Resources, Validation, Writing – review & editing. AO: Data curation, Investigation, Methodology, Project administration, Resources, Validation, Writing – review & editing. PS: Data curation, Formal analysis, Funding acquisition, Investigation, Methodology, Project administration, Resources, Validation, Visualization, Writing – review & editing. MM: Data curation, Funding acquisition, Investigation, Methodology, Project administration, Resources, Supervision, Validation, Visualization, Writing – review & editing. DA: Writing – original draft, Writing – review & editing, Conceptualization, Data curation, Formal analysis, Funding acquisition, Investigation, Methodology, Project administration, Resources, Software, Supervision, Validation, Visualization.
