# Supplementary material for: Gestation-dependent increase in cervicovaginal pro-inflammatory cytokines and cervical extracellular matrix proteins is associated with spontaneous preterm delivery within 2 weeks of index assessment in South African women
Source: Front Immunol. 2024 Aug 6;15:1377500. doi: 10.3389/fimmu.2024.1377500 (PMC11333255; doi:10.3389/fimmu.2024.1377500)
Supplement: Supplementary file 1 [file Image_1.pdf]

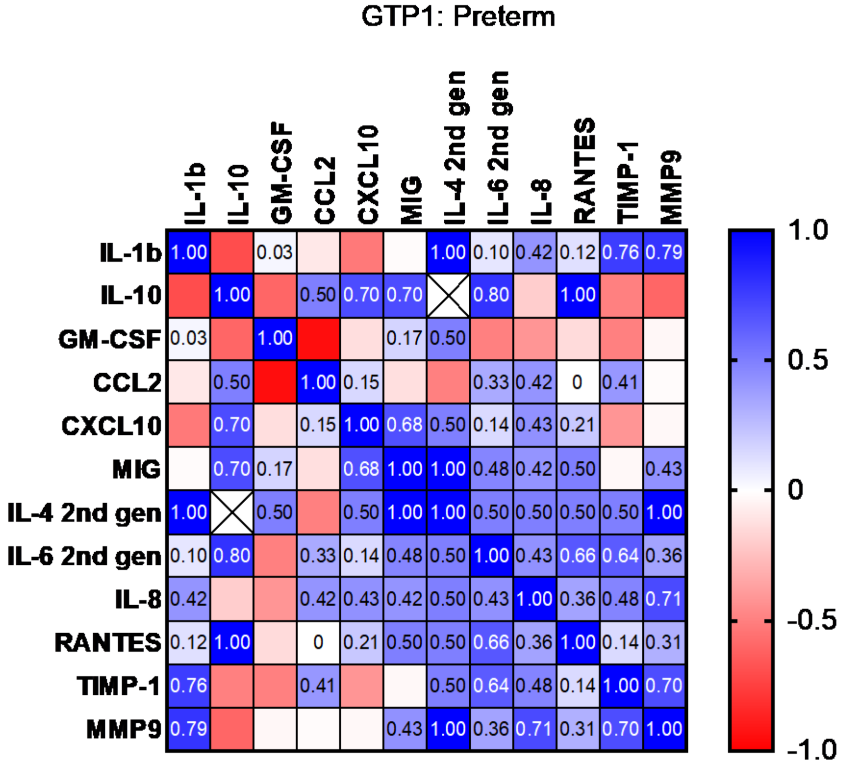
GTP1: Preterm
IL-1b
IL-10
GM-CSF
CCL2
CXCL10
MIG
IL-4 2nd gen
IL-6 2nd gen
IL-8
RANTES
TIMP-1
MMP9
1.0
0.5
0
-0.5
-1.0

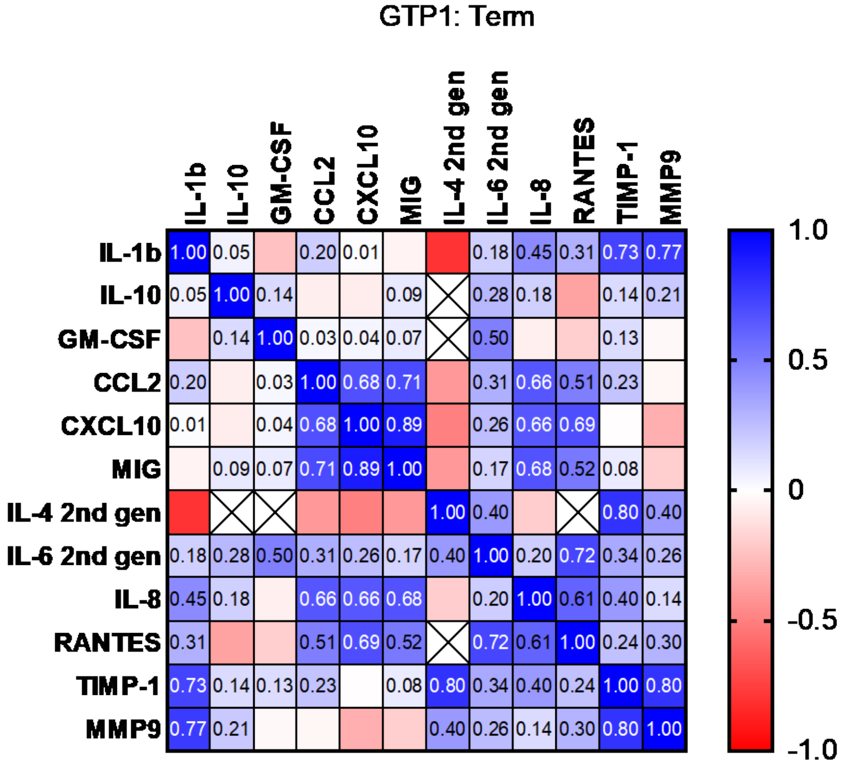
GTP1: Term
IL-1b
IL-10
GM-CSF
CCL2
CXCL10
MIG
IL-4 2nd gen
IL-6 2nd gen
IL-8
RANTES
TIMP-1
MMP9
1.0
0.5
0
-0.5
-1.0

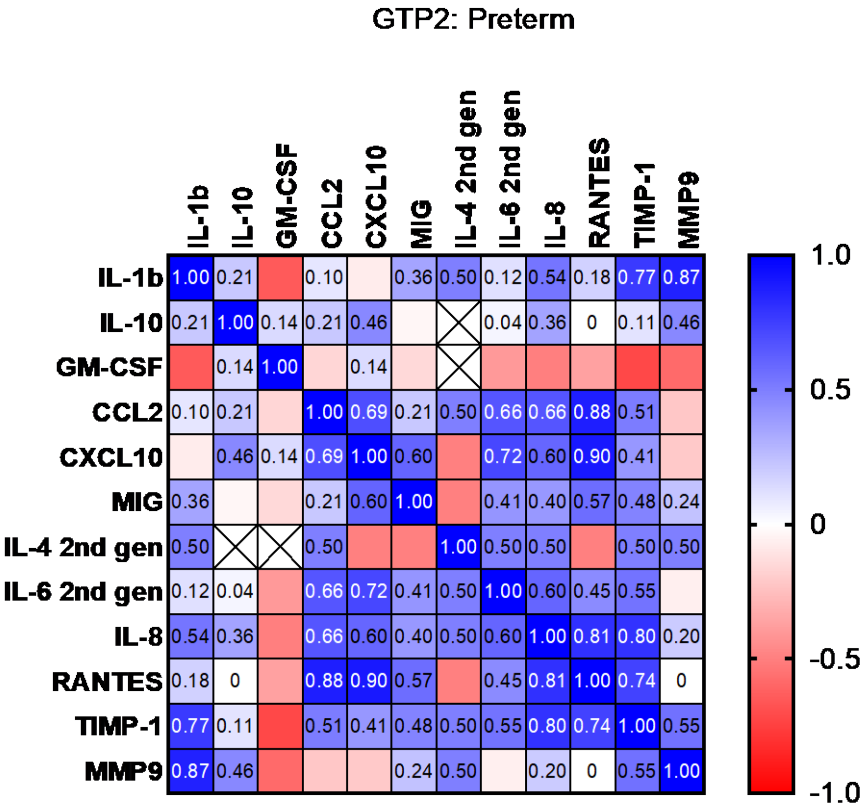
GTP2: Preterm
IL-1b
IL-10
GM-CSF
CCL2
CXCL10
MIG
IL-4 2nd gen
IL-6 2nd gen
IL-8
RANTES
TIMP-1
MMP9
IL-1b 1.00 0.21 0.10 0.36 0.50 0.12 0.54 0.18 0.77 0.87
IL-10 0.21 1.00 0.14 0.21 0.46 0.04 0.36 0 0.11 0.46
GM-CSF 0.14 1.00 0.14
CCL2 0.10 0.21 1.00 0.69 0.21 0.50 0.66 0.66 0.88 0.51
CXCL10 0.46 0.14 0.69 1.00 0.60 0.72 0.60 0.90 0.41
MIG 0.36 0.21 0.60 1.00 0.41 0.40 0.57 0.48 0.24
IL-4 2nd gen 0.50 0.50 1.00 0.50 0.50 0.50 0.50
IL-6 2nd gen 0.12 0.04 0.66 0.72 0.41 0.50 1.00 0.60 0.45 0.55
IL-8 0.54 0.36 0.66 0.60 0.40 0.50 0.60 1.00 0.81 0.80 0.20
RANTES 0.18 0 0.88 0.90 0.57 0.45 0.81 1.00 0.74 0
TIMP-1 0.77 0.11 0.51 0.41 0.48 0.50 0.55 0.80 0.74 1.00 0.55
MMP9 0.87 0.46 0.24 0.50 0.20 0 0.55 1.00
1.0
0.5
0
-0.5
-1.0

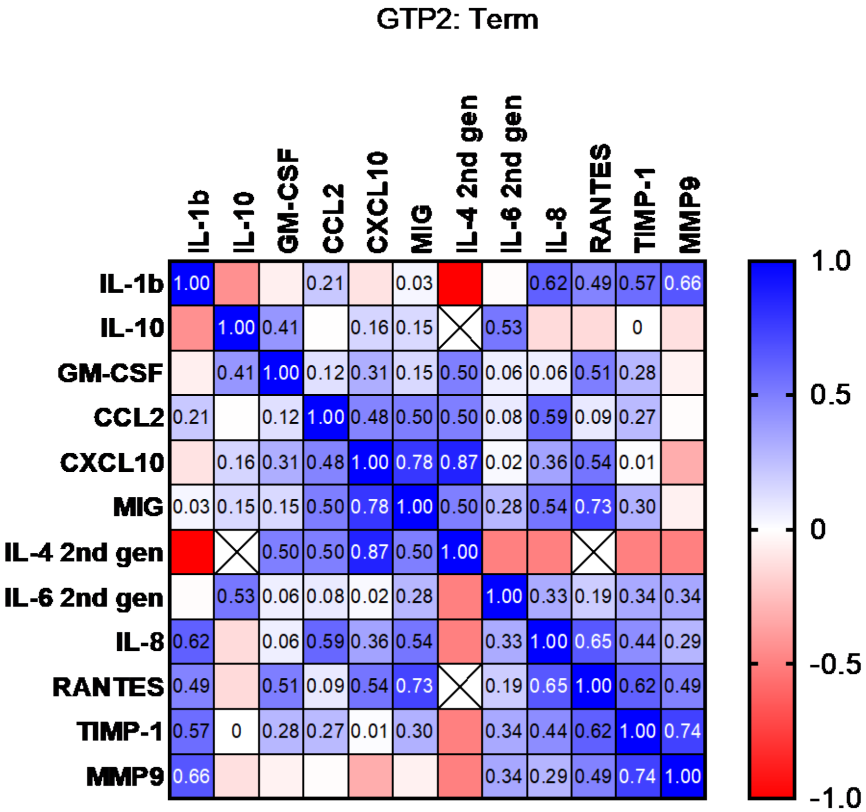
GTP2: Term
IL-1b
IL-10
GM-CSF
CCL2
CXCL10
MIG
IL-4 2nd gen
IL-6 2nd gen
IL-8
RANTES
TIMP-1
MMP9
IL-1b 1.00 0.21 0.03 0.62 0.49 0.57 0.66
IL-10 1.00 0.41 0.16 0.15 0.53 0
GM-CSF 0.41 1.00 0.12 0.31 0.15 0.50 0.06 0.06 0.51 0.28
CCL2 0.21 0.12 1.00 0.48 0.50 0.50 0.08 0.59 0.09 0.27
CXCL10 0.16 0.31 0.48 1.00 0.78 0.87 0.02 0.36 0.54 0.01
MIG 0.03 0.15 0.15 0.50 0.78 1.00 0.50 0.28 0.54 0.73 0.30
IL-4 2nd gen 0.50 0.50 0.87 0.50 1.00
IL-6 2nd gen 0.53 0.06 0.08 0.02 0.28 1.00 0.33 0.19 0.34 0.34
IL-8 0.62 0.06 0.59 0.36 0.54 0.33 1.00 0.65 0.44 0.29
RANTES 0.49 0.51 0.09 0.54 0.73 0.19 0.65 1.00 0.62 0.49
TIMP-1 0.57 0 0.28 0.27 0.01 0.30 0.34 0.44 0.62 1.00 0.74
MMP9 0.66 0.34 0.29 0.49 0.74 1.00
1.0
0.5
0
-0.5
-1.0

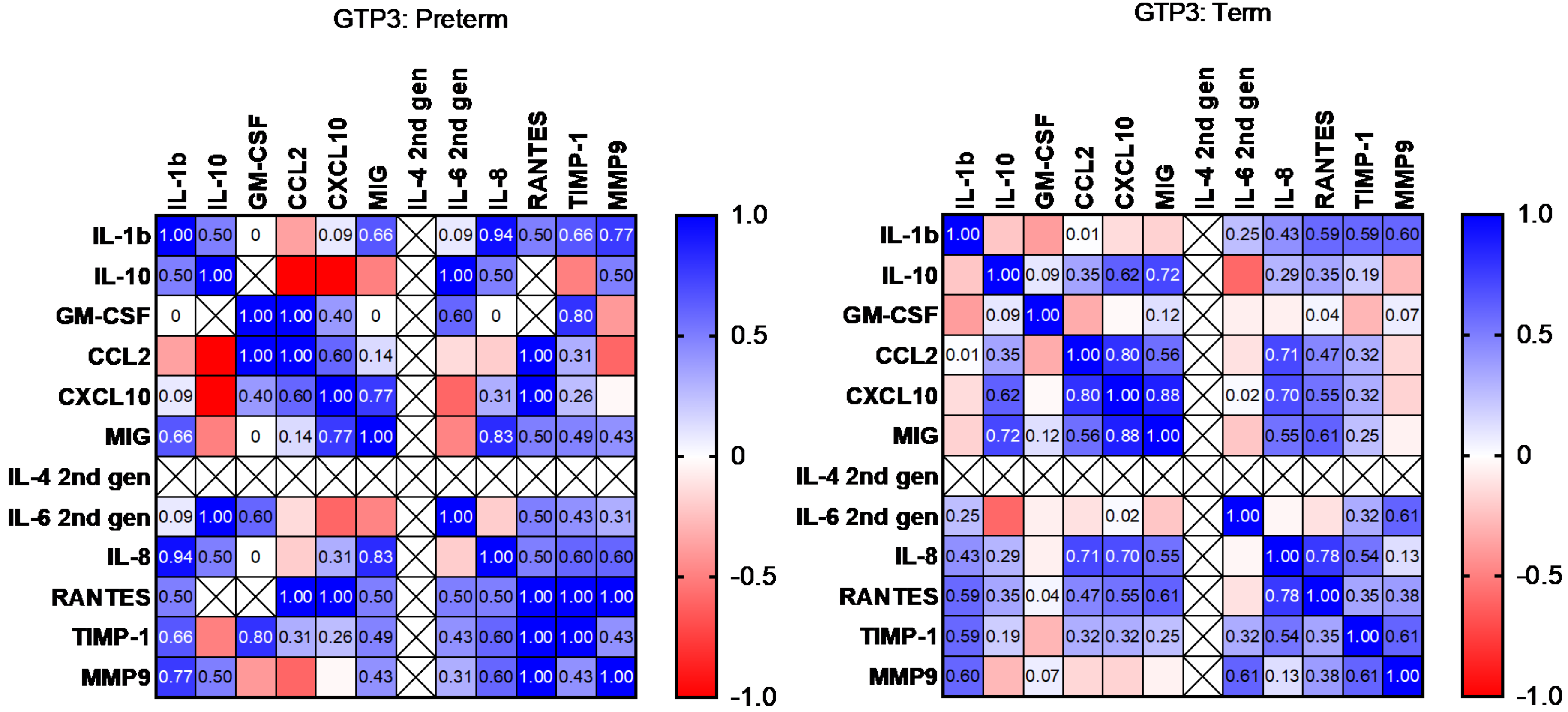

Supplementary Figure S1.

Correlation of cervicovaginal fluid cytokines and proteins associated with extracellular matrix remodelling at 20-22 weeks (GTP1), 26-28 weeks (GTP2), and 34-36 weeks (GTP3). CCL2, monocyte chemoattractant protein-1 (MCP-1); CXCL10, interferon gamma-induced protein 10 (IP-10); GM-CSF, granulocyte-macrophage colony-stimulating factor; IL, interleukin; MIG, monokine induced by interferon gamma (CXCL9); MMP-9, matrix-metalloproteinase-9; RANTES, regulated on activation, normal T cell expressed and secreted; TIMP-1, tissue inhibitor of metalloproteinase-1; GTP, gestational time point. Values in the coloured cells and right bar = , Spearman's correlation coefficient ranging from +1 (dark blue) to -1 (dark red); p-values are presented in Table S1.
