# Supplementary material for: Gestation-dependent increase in cervicovaginal pro-inflammatory cytokines and cervical extracellular matrix proteins is associated with spontaneous preterm delivery within 2 weeks of index assessment in South African women
Source: Front Immunol. 2024 Aug 6;15:1377500. doi: 10.3389/fimmu.2024.1377500 (PMC11333255; doi:10.3389/fimmu.2024.1377500)
Supplement: Supplementary file 2 [file Table_1.docx]

**Supplementary material**

**Supplementary Table S1.** Correlation of cervicovaginal fluid cytokines and proteins associated with extracellular matrix remodelling according to birth outcome

|  | **Spearman *⍴*** | ***p* value** |
| --- | --- | --- |
| **GTP1 (20-22 weeks)** | | |
| **Preterm:** |  |  |
| IL-1β v MMP-9 | 0.794 | 0.009 |
| IL-1β v TIMP-1 | 0.758 | 0.015 |
| GM-CSF v CCL2 | -0.950 | 0.0004 |
| IL-8 v MMP-9 | 0.709 | 0.027 |
| CXCL10 v MIG | 0.685 | 0.035 |
| TIMP-1 v MMP-9 | 0.697 | 0.031 |
| **Term:** |  |  |
| IL-1β v IL-8 | 0.454 | 0.020 |
| IL-1β v TIMP-1 | 0.733 | <0.0001 |
| IL-1β v MMP-9 | 0.766 | <0.0001 |
| IL-8 v RANTES | 0.610 | 0.030 |
| IL-8 v TIMP-1 | 0.400 | 0.043 |
| IL-8 v MIG | 0.677 | 0.0001 |
| CCL2 v CXCL10 | 0.683 | 0.0002 |
| CCL2 v MIG | 0.709 | <0.0001 |
| CCL2 v IL-8 | 0.660 | 0.0002 |
| CXCL10 v MIG | 0.894 | <0.0001 |
| CXCL10 v IL-8 | 0.656 | 0.001 |
| CXCL10 v RANTES | 0.692 | 0.011 |
| TIMP1 v MMP-9 | 0.796 | <0.0001 |
| **GTP2 (26-28 weeks)** | | |
| **Preterm:** |  |  |
| IL-1β v TIMP-1 | 0.769 | 0.005 |
| IL-1β v MMP-9 | 0.867 | 0.001 |
| IL-6 v IL-8 | 0.601 | 0.043 |
| IL-6 v CCL2 | 0.664 | 0.022 |
| IL-6 v CXCL10 | 0.720 | 0.011 |
| IL-8 v CCL2 | 0.665 | 0.016 |
| IL-8 v RANTES | 0.452 | 0.022 |
| IL-8 v TIMP-1 | 0.802 | 0.002 |
| IL-8 v CXCL10 | 0.599 | 0.034 |
| CCL2 v CXCL10 | 0.692 | 0.011 |
| CCL2 v RANTES | 0.881 | 0.007 |
| CXCL10 v MIG | 0.604 | 0.032 |
| CXCL10 v RANTES | 0.905 | 0.005 |
| GM-CSF v TIMP-1 | -0.721 | 0.023 |
| RANTES v TIMP-1 | 0.738 | 0.046 |
| **Term:** |  |  |
| IL-1β v TIMP-1 | 0.569 | 0.002 |
| IL-1β v MMP-9 | 0.661 | 0.0001 |
| IL-1β v IL-8 | 0.621 | 0.0005 |
| IL-8 v CCL2 | 0.593 | 0.001 |
| IL-8 v RANTES | 0.653 | 0.014 |
| IL-8 v TIMP-1 | 0.444 | 0.020 |
| IL-8 v MIG | 0.537 | 0.004 |
| MIG v RANTES | 0.732 | 0.004 |
| MIG v CXCL10 | 0.782 | <0.0001 |
| MIG v CCL2 | 0.505 | 0.007 |
| CXCL10 v CCL2 | 0.482 | 0.013 |
| TIMP-1 v RANTES | 0.622 | 0.020 |
| TIMP-1 v MMP-9 | 0.744 | <0.0001 |
| **GTP3 (34-36 weeks)** | | |
| **Preterm:** |  |  |
| IL-1β v IL-8 | 0.943 | 0.017 |
| **Term:** |  |  |
| IL-1β v IL-8 | 0.432 | 0.040 |
| IL-1β v MMP-9 | 0.600 | 0.005 |
| IL-1β v TIMP-1 | 0.590 | 0.005 |
| IL-1β v RANTES | 0.586 | 0.024 |
| IL-6 v MMP-9 | 0.609 | 0.024 |
| IL-8 v TIMP-1 | 0.538 | 0.012 |
| IL-8 v RANTES | 0.775 | 0.001 |
| IL-10 v CXCL10 | 0.622 | 0.020 |
| IL-10 v MIG | 0.718 | 0.003 |
| CCL2 v CXCL10 | 0.804 | <0.0001 |
| CCL2 v MIG | 0.563 | 0.005 |
| CCL2 v IL-8 | 0.714 | 0.0001 |
| CXCL10 v MIG | 0.878 | <0.0001 |
| CXCL10 v IL-8 | 0.695 | 0.0003 |
| CXCL10 v RANTES | 0.547 | 0.037 |
| MIG v IL-8 | 0.551 | 0.006 |
| MIG v RANTES | 0.614 | 0.017 |
| TIMP-1 v MMP-9 | 0.606 | 0.005 |

*CCL2*, monocyte chemoattractant protein-1 (MCP-1); *CXCL10*, interferon gamma-induced protein 10 (IP-10); *GM-CSF*, granulocyte-macrophage colony-stimulating factor; *IL*, interleukin; *MIG*, monokine induced by interferon gamma *(CXCL9)*; *MMP-9*, matrix-metalloproteinase-9; *RANTES*, regulated on activation, normal T cell expressed and secreted; *TIMP-1*, tissue inhibitor of metalloproteinase-1; *GTP*, gestational time point; *⍴*, correlation coefficient (rho); *p*, probability

**Supplementary Table S2.** Correlation of cervicovaginal fluid cytokines and proteins associated with extracellular matrix remodelling in the total population (regardless of birth outcome)

|  | **Spearman *r*** | ***p* value** |
| --- | --- | --- |
| **GTP1 (20-22 weeks)** | | |
| IL-1β v MMP-9 | 0.784 | <0.0001 |
| IL-1β v TIMP-1 | 0.713 | <0.0001 |
| IL-1β v IL-8 | 0.575 | 0.0001 |
| CCL2 v CXCL10 | 0.515 | 0.002 |
| CCL2 v IL-8 | 0.512 | 0.001 |
| CXCL10 v MIG | 0.820 | <0.0001 |
| CXCL10 v IL-8 | 0.557 | 0.001 |
| CXCL10 v RANTES | 0.590 | 0.004 |
| IL-6 v RANTES | 0.669 | 0.015 |
| IL-6 v TIMP-1 | 0.479 | 0.015 |
| IL-8 v MIG | 0.628 | <0.0001 |
| IL-8 v RANTES | 0.443 | 0.039 |
| IL-8 v TIMP-1 | 0.427 | 0.008 |
| TIMP-1 v MMP-9 | 0.756 | <0.0001 |
|  | **GTP2 (26-28 weeks)** |  |
| IL-1β v IL-8 | 0.644 | <0.0001 |
| IL-1β v IL-10 | -0.390 | 0.044 |
| IL-1β v MMP-9 | 0.796 | <0.0001 |
| IL-1β v TIMP-1 | 0.607 | <0.0001 |
| GM-CSF v IL-10 | 0.402 | 0.042 |
| CCL2 v CXCL10 | 0.699 | <0.0001 |
| CCL2 v MIG | 0.426 | 0.006 |
| CCL2 v IL-6 | 0.390 | 0.036 |
| CCL2 v IL-8 | 0.547 | 0.0003 |
| CCL2 v RANTES | 0.471 | 0.027 |
| CCL2 v TIMP-1 | 0.329 | 0.038 |
| CXCL10 v MIG | 0.712 | <0.0001 |
| CXCL10 v IL-6 | 0.394 | 0.035 |
| CXCL10 v IL-8 | 0.435 | 0.006 |
| CXCL10 v RANTES | 0.675 | 0.001 |
| MIG vs IL-8 | 0.508 | 0.001 |
| MIG vs IL-6 | 0.385 | 0.039 |
| MIG v RANTES | 0.636 | 0.001 |
| MIG v TIMP-1 | 0.383 | 0.015 |
| IL-6 v IL-8 | 0.446 | 0.015 |
| IL-6 v TIMP-1 | 0.406 | 0.029 |
| IL-8 v RANTES | 0.641 | 0.001 |
| IL-8 v TIMP-1 | 0.582 | <0.0001 |
| IL-8 v MMP-9 | 0.406 | 0.009 |
| RANTES v TIMP-1 | 0.609 | 0.003 |
| TIMP-1 v MMP-9 | 0.692 | <0.0001 |
| **GTP3 (34-36 weeks)** | | |
| IL-1β v MMP-9 | 0.733 | <0.0001 |
| IL-1β v TIMP-1 | 0.646 | 0.0003 |
| IL-1β v IL-8 | 0.621 | 0.0003 |
| IL-1β v RANTES | 0.589 | 0.010 |
| CCL2 v CXCL10 | 0.777 | <0.0001 |
| CCL2 v MIG | 0.510 | 0.005 |
| CCL2 v RANTES | 0.538 | 0.021 |
| CCL2 v IL-8 | 0.578 | 0.001 |
| CXCL10 v MIG | 0.861 | <0.0001 |
| CXCL10 v IL-8 | 0.636 | 0.0003 |
| CXCL10 v RANTES | 0.600 | 0.008 |
| MIG v IL-8 | 0.639 | 0.0002 |
| MIG v RANTES | 0.662 | 0.003 |
| IL-6 v MMP-9 | 0.626 | 0.003 |
| IL-8 v RANTES | 0.781 | 0.0001 |
| IL-8 v TIMP-1 | 0.620 | 0.0006 |
| IL-8 v MMP-9 | 0.398 | 0.044 |
| TIMP-1 v MMP-9 | 0.659 | 0.0002 |

*CCL2*, monocyte chemoattractant protein-1 (MCP-1); *CXCL10*, interferon gamma-induced protein 10 (IP-10); *GM-CSF*, granulocyte-macrophage colony-stimulating factor; *IL*, interleukin; *MIG*, monokine induced by interferon gamma *(CXCL9)*; *MMP-9*, matrix-metalloproteinase-9; *RANTES*, regulated on activation, normal T cell expressed and secreted; *TIMP-1*, tissue inhibitor of metalloproteinase-1; *GTP*, gestational time point; *r*, correlation coefficient; *P*, probability
